# Exploring the suitability of the CHIME framework in Occupational Therapy for people living with mild dementia: a qualitative study including perspectives from the Black Caribbean British experience

**DOI:** 10.1002/alz70858_107678

**Published:** 2025-12-25

**Authors:** Lesley Garcia

**Affiliations:** ^1^ University of Nottingham, Nottingham, United Kingdom

## Abstract

**Background:**

Black Caribbean British people living with dementia show higher rates of dementia yet poorer access to dementia care services (Mukadam et al., 2023). In the UK, the use of recovery approaches with people living with dementia has increasingly been endorsed along with a call for an emphasis on dementia research focusing on early diagnosis and interventions (West et al., 2022; Handley et al., 2024). The CHIME (Connectedness, Hope and Optimism, Identity, Meaning in life, Empowerment) framework (Leamy et al., 2011) operationalised the recovery approach in this study. The World Health Organisation has identified the profession of occupational therapy (OT) as one of the key health disciplines with the professional competence to provide skilled therapeutic services to people living with dementia at all stages of the disease (Jeon et al., 2023).

**Methods:**

In order to enquire into the suitability of applying the CHIME framework with the population of people living with mild dementia with a special focus on Black Caribbean British people living with mild dementia an exploratory descriptive qualitative (EDQ) study (Hunter, Mccallum and Howes, 2019) was conducted. (OTs) from both White British and Black Caribbean British heritages were recruited, consented and participated in semi‐structured interviews of 60–90 minutes each. Reflexive thematic analysis (RTA) was utilised following the process outlined by Braun and Clarke (2019).

**Results:**

High information power (Malterud et al., 2016) of the sample group (*N* = 9) generated a rich and descriptive dataset comprised of 14 hours of dialogue and 111,000 words in verbatim transcriptions. RTA led to the development of four overarching themes: CHIME Model is Core to OT Dementia Practice yet Tacit; OT Practitioners Recognise the Limitations of Cultural Competence; Operationalising CHIME in Dementia Practice through Transformative Learning; and Flexible and Adaptive use of Technology in Dementia OT practice.

**Conclusions:**

The findings of the study suggest that while OTs already practice with a tacit understanding of recovery (CHIME) principles, a more explicitly CHIME‐informed approach could strengthen therapeutic relationships and enhance trust and engagement. This is particularly indicated among Black Caribbean British people living with mild dementia. OT's would welcome post‐registration continuing education to facilitate this skillset.

